# Identification of Key Determinants of Staphylococcus aureus Vaginal Colonization

**DOI:** 10.1128/mBio.02321-19

**Published:** 2019-12-24

**Authors:** Liwen Deng, Katrin Schilcher, Lindsey R. Burcham, Jakub M. Kwiecinski, Paige M. Johnson, Steven R. Head, David E. Heinrichs, Alexander R. Horswill, Kelly S. Doran

**Affiliations:** a Department of Immunology and Microbiology, University of Colorado School of Medicine, Aurora, Colorado, USA; b Department of Cell and Molecular Biology, San Diego State University, San Diego, California, USA; c Next Generation Sequencing Core, The Scripps Research Institute, La Jolla, California, USA; d Department of Microbiology and Immunology, University of Western Ontario, London, Ontario, Canada; e Department of Veterans Affairs Eastern, Colorado Healthcare System, Aurora, Colorado, USA; University of Rochester

**Keywords:** MRSA, RNA sequencing, *Staphylococcus aureus*, fibrinogen, iron, vaginal colonization

## Abstract

Staphylococcus aureus is an important pathogen responsible for nosocomial and community-acquired infections in humans, and methicillin-resistant S. aureus (MRSA) infections have continued to increase despite widespread preventative measures. S. aureus can colonize the female vaginal tract, and reports have suggested an increase in MRSA infections in pregnant and postpartum women as well as outbreaks in newborn nurseries. Currently, little is known about specific factors that promote MRSA vaginal colonization and subsequent infection. To study S. aureus colonization of the female reproductive tract in a mammalian system, we developed a mouse model of S. aureus vaginal carriage and demonstrated that both hospital-associated and community-associated MRSA isolates can colonize the murine vaginal tract. Immunohistochemical analysis revealed an increase in neutrophils in the vaginal lumen during MRSA colonization. Additionally, we observed that a mutant lacking fibrinogen binding adhesins exhibited decreased persistence within the mouse vagina. To further identify novel factors that promote vaginal colonization, we performed RNA sequencing to determine the transcriptome of MRSA growing *in vivo* during vaginal carriage at 5 h, 1 day, and 3 days postinoculation. Over 25% of the bacterial genes were differentially regulated at all time points during colonization compared to laboratory cultures. The most highly induced genes were those involved in iron acquisition, including the Isd system and siderophore transport systems. Mutants deficient in these pathways did not persist as well during *in vivo* colonization. These results reveal that fibrinogen binding and the capacity to overcome host nutritional limitation are important determinants of MRSA vaginal colonization.

## Abstract (precis)

Staphylococcus aureus is an opportunistic pathogen able to cause a wide variety of infections in humans. Recent reports have suggested an increasing prevalence of MRSA in pregnant and postpartum women, coinciding with the increased incidence of MRSA infections in neonatal intensive care units (NICUs) and newborn nurseries. Vertical transmission from mothers to infants at delivery is a likely route of MRSA acquisition by the newborn; however, essentially nothing is known about host and bacterial factors that influence MRSA carriage in the vagina. Here, we established a mouse model of vaginal colonization and observed that multiple MRSA strains can persist in the vaginal tract. Additionally, we determined that MRSA interactions with fibrinogen and iron uptake can promote vaginal persistence. This study is the first to identify molecular mechanisms which govern vaginal colonization by MRSA, the critical initial step preceding infection and neonatal transmission.

## INTRODUCTION

Staphylococcus aureus is a commensal of approximately 20% of the healthy adult population (1) and an opportunistic bacterial pathogen able to cause a wide variety of infections ranging in severity from superficial skin lesions to more serious invasive and life-threatening infections, such as endocarditis and septicemia. The prevalence of S. aureus infections has increased due to higher rates of colonization and immunosuppressive conditions, greater use of surgical implants, and dramatic increases in antibiotic resistance (2, 3). Compared to antibiotic-susceptible strains, methicillin-resistant S. aureus (MRSA) infections exhibit elevated mortality rates, require longer hospital stays, and exert a higher financial burden on patients and health care institutions (4). Over the past 20 years, MRSA strains have expanded from health care settings and began infecting otherwise healthy individuals in the community (“community-associated” MRSA [CA-MRSA]). In contrast to health care-associated MRSA (HA-MRSA), CA-MRSA strains are more virulent and can spread rapidly among healthy individuals (5). USA300 isolates are the most problematic lineage of CA-MRSA that have emerged and clonally expanded across the United States, reaching epidemic levels in many hospital settings (6, 7).

Methicillin-susceptible S. aureus and MRSA possess many virulence factors that promote bacterial persistence and invasive infections in different host sites. These virulence factors include cell wall-anchored surface proteins that facilitate S. aureus adherence to and invasion of host cells (8), proteases that modulate the host immune response to the bacterium (9), as well as pore-forming toxins such as alpha toxin and the bicomponent leukocidins that lyse host cells (10). The expression of these various virulence determinants is dependent on factors such as growth rate, the availability of certain nutrients, host interactions, and the presence of antimicrobial compounds (8, 11–13).

Nasal carriage is known to be a risk factor for S. aureus infections in both the hospital and the community, with individuals often being infected with the strain that they carry (14). S. aureus can colonize the moist squamous epithelium in the anterior nares (15, 16), a process which depends upon specific interactions between bacterial cell adhesins and epithelial cell ligands. Two S. aureus surface proteins, clumping factor B (ClfB) and iron regulated surface determinant A (IsdA), have been strongly implicated in nasal colonization. Both ClfB and IsdA were shown to promote adhesion to nasal epithelium *in vitro* (17) and colonization of the nares of rodents (18, 19) and, in the case of ClfB, humans (20). ClfB is a member of a family of proteins that are structurally related to clumping factor A (ClfA), the archetypal fibrinogen (Fg) binding protein of S. aureus. ClfB has been shown to bind Fg, as well as cytokeratin 10, by the “dock, lock, and latch” mechanism first defined for the Fg binding proteins SdrG and ClfA (21, 22). Additional surface proteins shown to contribute to bacterial attachment to nasal epithelial cells *in vitro* include S. aureus surface protein G (SasG) and the serine-aspartate repeat proteins SdrC and SdrD (23).

While a ubiquitous colonizer of the skin and mucous membranes, S. aureus, including antibiotic sensitive and resistant strains, has also been reported to colonize the vagina in up to 22% of pregnant women (24–29). A study that examined MRSA colonization showed that out of 5,732 mothers, 3.5% were colonized by MRSA in the genital tract during pregnancy (24). Another recent study of 1,834 mothers showed that 4.7% were colonized vaginally by multidrug-resistant S. aureus (30). Reports have suggested an increasing prevalence in the USA300 lineage of MRSA in colonization of pregnant and postpartum women, coinciding with the increased incidence in NICUs and in newborn nurseries. The most common presentations of MRSA disease in pregnant and postpartum women include skin and soft tissue infections and puerperal mastitis. Neonatal MRSA invasive disease presentations include bacteremia, meningitis, and urinary tract infections (31–36). MRSA outbreaks in NICUs can be difficult to control and have been associated with significant morbidity and mortality (33). Because S. aureus vaginal carriage in mothers, as detected by rectovaginal swabbing, is significantly correlated with S. aureus colonization of their newborns, vertical transmission from mothers to infants at delivery has been proposed as a possible mechanism of neonatal CA-MRSA acquisition (30, 37), and while it is clear that S. aureus and MRSA can colonize the vaginal tract during pregnancy, essentially nothing is known about specific bacterial factors that promote vaginal persistence.

In this study, we have adapted a murine model of vaginal colonization by group B Streptococcus (GBS) (38) to investigate MRSA vaginal colonization. We determined that divergent MRSA strains, CA-MRSA USA300 and HA-MRSA252, can persist within the mouse vaginal tract and that three mouse strains, CD-1, C57BL/6, and BALB/c, can be colonized with MRSA. We detected fluorescent MRSA in the vaginal lumen as well as in cervical and uterine tissues of colonized mice, and immunohistochemical staining showed an increase in neutrophils in colonized mice compared to naive mice. We found that a MRSA strain lacking fibrinogen binding surface adhesins was attenuated in both *in vitro* and *in vivo* models of vaginal colonization. Lastly, RNA sequencing analysis of bacteria growing *in vivo* revealed the importance of iron homeostasis in promoting MRSA persistence within the mouse vagina. Mutant USA300 strains lacking the siderophore transporter FhuCBG or the cell surface hemoglobin receptor IsdB were significantly attenuated in their ability to colonize the vaginal tract *in vivo*.

## RESULTS

### MRSA colonization of the reproductive tract.

**FIG 1:**
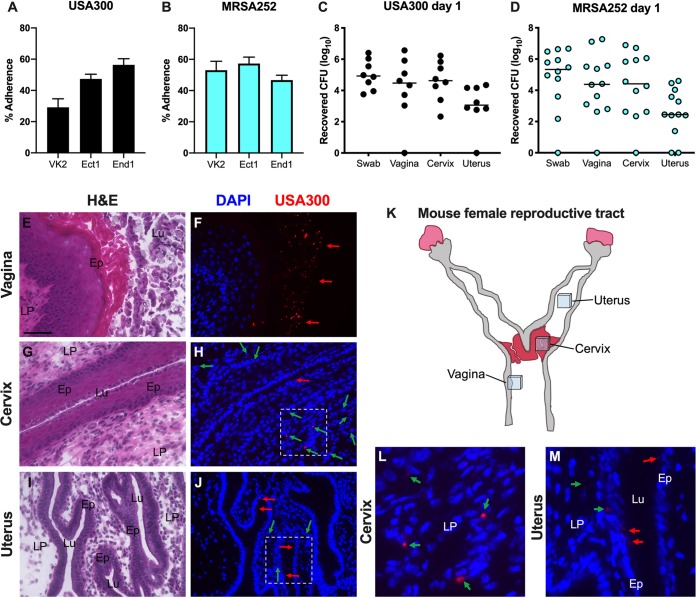
Modeling MRSA vaginal colonization. (A and B) Adherence of USA300 (A) and MRSA252 (B) to human vaginal (VK2), ectocervical (Ect1), and endocervical (End1) endothelial cells. Experiments were performed three times in triplicate, and error bars represent the standard deviations (SDs); the results of a representative experiment are shown. (C and D) CFU counts from vaginal swabs, vagina, cervix, and uterus recovered 1 day postinoculation with USA300 (C) or MRSA252 (D). Horizontal lines represent median CFU counts. (E to M) Mice were colonized with DsRed expressing USA300. One day postinoculation, the female reproductive tract tissues were harvested, and 6-μm coronal sections of the vagina (E and F), cervix (G, H, and L), and uterus (I, J, and M) were either stained with H&E for bright-field microscopy (E, G, I) or labeled with DAPI for imaging with an epifluorescence microscope to visualize nuclei and USA300 (F, H, J, L, and M). (K) A schematic of the mouse female reproductive tract; the locations of the tissue sections taken for histological analysis are shown. The areas highlighted in panels H and J are expanded in panels L and M. USA300 cells in the lumen (Lu) and epithelial layer (Ep) of tissues are indicated with red arrows, and USA300 cells within the lamina propia (LP) are indicated with green arrows. (E) Scale bar = 100 μm.

To characterize the ability of MRSA to attach to epithelial cells of the lower female reproductive tract, we performed quantitative adherence assays with community-associated USA300 strain LAC (39) and hospital-acquired strain MRSA252 (40), as described in reference 41 and in Materials and Methods. An inoculum of 10^5^ CFU/well (multiplicity of infection [MOI], 1) was added to confluent monolayers of immortalized human vaginal (VK2), ectocervical (Ect1), and endocervical (End1) epithelial cells. Following a 30-minute incubation, the cells were washed to remove all nonadherent bacteria. Data are expressed as the percent recovered cell-associated MRSA relative to the initial inoculum. Both strains exhibited substantial adherence to all three cell lines, ranging from 30 to 57% of the original inoculum (Fig. 1A and B). In contrast, MRSA adherence to human nasal epithelial cells has been reported to be 10% of the original inoculum (42).

Next, we assessed the ability of both MRSA strains to initiate colonization of the murine vaginal tract. We have previously demonstrated for a murine model of GBS vaginal colonization that mice inoculated at the proestrus stage of the estrous cycle were colonized with GBS longer than were mice inoculated at any other stage (38). Therefore, we synchronized 8-week old female CD-1 mice in proestrus by treating with 17β-estradiol 1 day before inoculation with 10^7^ CFU of either USA300 or MRSA252. The next day, the vaginal lumen was swabbed, and then we euthanized the animals and collected the vagina, cervix, and uterus from each mouse to quantify the bacterial load. The total CFU from the swab or tissue homogenates was determined by plating on S. aureus CHROMagar supplemented with cefoxitin. Both strains of MRSA were recovered from the majority of mice at 1 day postinoculation in all tissues, and the CFU counts recovered from the swab were similar to the total CFU counts from the vaginal tissue homogenates (Fig. 1C and D). This level and range in recovered CFU are similar to what we have observed using this mouse model for GBS colonization (38). In a subsequent experiment, mice were inoculated with USA300 expressing a fluorescent DsRed protein, and we harvested the female reproductive tract at 1 day postcolonization for histological analysis. We made coronal sections of these tissues and performed hematoxylin and eosin (H&E) staining to examine overall tissue morphology (Fig. 1E, G, and I) and fluorescence microscopy to visualize USA300 (Fig. 1F, H, J, L, and M) on serial sections. We observed numerous red fluorescent bacteria contained within the lumen of the vagina (red arrows) (Fig. 1F). We could also see MRSA cells attached to the epithelium of the cervix and the uterine glands, as well as within the lamina propria of those organs (green arrows) (Fig. 1H, J, L, and M).

### MRSA vaginal persistence and host response.

**FIG 2:**
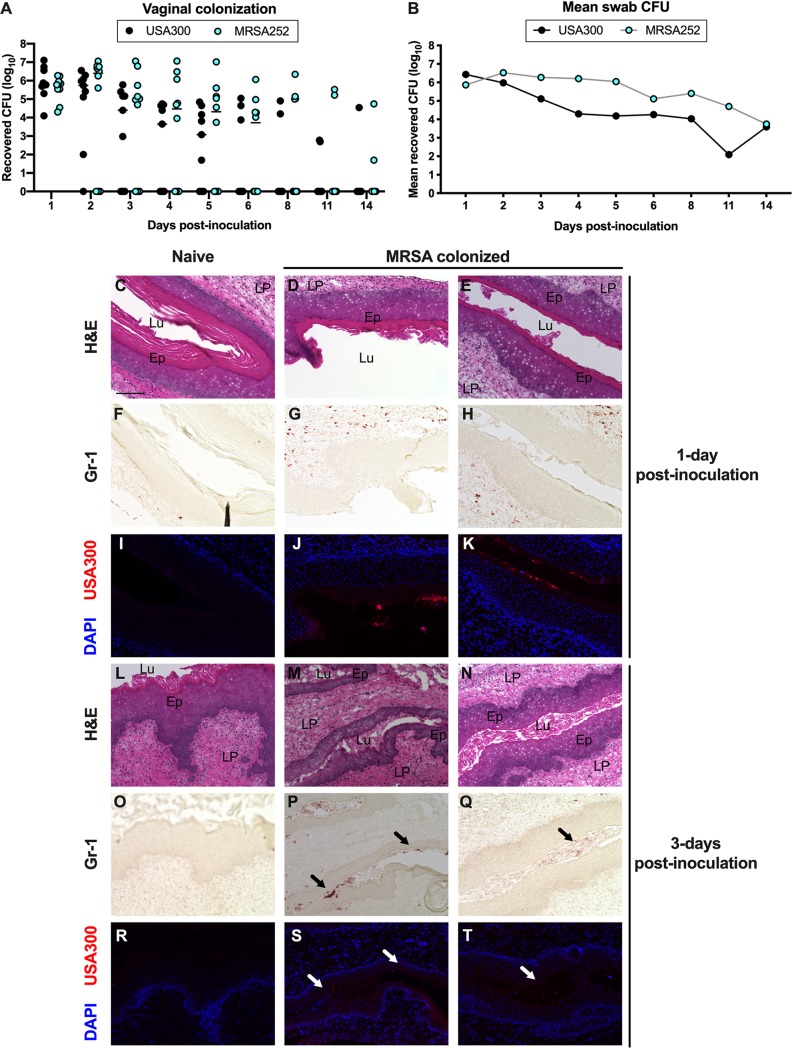
MRSA vaginal persistence and host response. (A and B) USA300 and MRSA252 persistence within the CD-1 mouse vaginal tract. CFU counts for individual mice (A) and mean recovered CFU from vaginal swabs (B) were monitored for 14 days. (A) Horizontal lines represent median CFU counts. (C to T) Histology of the mouse vagina during MRSA colonization. Mice were pretreated with 17β-estradiol and either remained naive (C, F, I, L, O, and R) or were inoculated with 10^7^ CFU of USA300 (D, E, G, H, J, K, M, N, P, Q, S, and T). Six-micrometer serial sections were stained with H&E (C to E and L to N), labeled with an antibody against Gr-1 (F to H and O to Q), or labeled with DAPI for fluorescence microscopy (I to K and R to T). (C) Scale bar = 100 μm. The lamina propria (LP), epithelium (E), and lumen (Lu) of the vaginal sections are labeled in the H&E images. Black arrows in P and Q indicate neutrophils and white arrows in S and T indicate MRSA in the vaginal lumen.

To assess vaginal persistence, mice were colonized with USA300 or MRSA252 and swabbed to determine the bacterial load over time. We recovered similar CFU from mice colonized with either MRSA strain, and we observed that both strains exhibited similar persistence within the mouse vagina. While all mice were initially highly colonized by both MRSA strains, some remained highly colonized, while MRSA was cleared from other mice (Fig. 2A and B). We also assessed USA300 vaginal colonization for multiple mouse strains and observed the highest mean CFU from BALB/c mice, while C57BL/6 and CD-1 mice were colonized to a lower level (see Fig. S1 in the supplemental material). Furthermore, MRSA was cleared more rapidly from the vaginal tract of CD-1 mice and persisted the longest in BALB/c mice (Fig. S1).

10.1128/mBio.02321-19.1 FIG S1 USA300 vaginal colonization in different mouse strains. (A and B) CD-1, C57BL/6, and BALB/c mice were inoculated with 10^7^ CFU of USA300, and the percentage of mice colonized (A) and the mean vaginal swab CFU (B) were monitored for 9 days. Download FIG S1, TIF file, 0.4 MB. Copyright © 2019 Deng et al. 2019 Deng et al. This content is distributed under the terms of the Creative Commons Attribution 4.0 International license.

As we observed eventual clearance of MRSA from the vaginal tract, we examined the presence of neutrophils in the vaginal tissue of mice colonized with MRSA compared to that in naive mice. Previous studies have shown that neutrophils respond to vaginal colonization by pathogenic *Streptococcus* species, namely, GBS and Streptococcus pyogenes (group A *Streptococcus* [GAS]), and that neutrophils contribute to host defense and ultimate bacterial clearance (43–45). To visualize neutrophils during colonization by MRSA, we collected vaginal tissues from mice at 1 day and 3 days postinoculation with DsRed expressing USA300 and made serial sections for H&E staining, labeling with an antibody against the neutrophil marker Gr-1, and fluorescence microscopy. H&E analysis showed that there were no obvious differences in the morphologies of the vaginal lumen between naive and colonized mice (Fig. 2C to E, and L to N). We observed very few Gr-1-positive cells in the tissue sections from naive mice (Fig. 2F and O). In contrast to those from naive mice, the tissue sections from mice colonized with USA300 for 1 day contained numerous neutrophils within the vaginal lamina propria (Fig. 2G and H). At 3 days postinoculation, we detected neutrophils within the vaginal lumen (black arrows) (Fig. 2P and Q). While we could visualize many MRSA cells in the vaginal lumen at 1 day postinoculation, there were much fewer fluorescent bacteria at 3 days postinoculation (Fig. 2J, K, S, and T). Interestingly, for the tissues collected 3 days postinoculation, the brightest MRSA signals were in the same areas as the strongest Gr-1 staining (white arrows).

### Adherence to fibrinogen impacts MRSA vaginal colonization.

**FIG 3:**
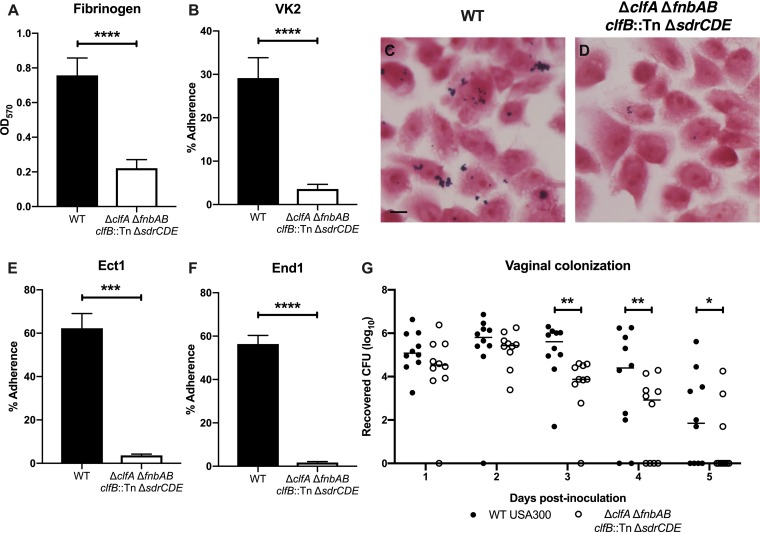
Adherence to fibrinogen impacts MRSA vaginal colonization. (A) Adherence of WT USA300 and the Fg adhesin mutant to Fg. (B to D) Adherence to VK2 cells. Monolayers of VK2 cells were inoculated with WT USA300 or the Fg adhesin mutant for a quantitative adherence assay (B) or Gram stains (C and D). (C) Scale bar = 10 μm. (E and F) Adherence to Ect1 (E) and End1 (F) epithelial cells. Adherence assays were performed at least three times in triplicate, and error bars represent SDs; the results of a representative experiment are shown. (G) WT USA300 and the Fg adhesin cocolonization. (A, B, E, and F) Statistical analysis using an unpaired *t* test. (G) Two-way analysis of variance (ANOVA) with Sidak’s multiple-comparison test. *, *P* < 0.05; ***, *P* < 0.0005; ****, *P* < 0.00005.

In a previous study, we demonstrated that GBS Fg binding contributed to vaginal persistence (46). Also, several studies have shown the importance of S. aureus interactions with extracellular matrix components, including Fg, in colonization and disease progression (47–50). USA300 binding to Fg is primarily mediated by the four sortase-anchored surface adhesins ClfA, ClfB, FnbA, and FnbB (8, 47, 51). The serine-aspartate adhesins SdrC, SdrD, and SdrE are in the same protein family as ClfA/B (52) and have been reported to bind nasal epithelia (23). To eliminate these adherence functions, a USA300 strain was engineered in which all of these adhesins were deleted or disrupted by incorporating four separate mutations (Δ*clfA clfB*::Tn Δ*fnbAB sdrCDE*::Tet; here called the “Fg adhesin mutant”). Compared to wild-type (WT) USA300, the Fg adhesin mutant was significantly less adherent to Fg (Fig. 3A). Quantitative adherence assays showed that the fibrinogen adhesin mutant exhibited decreased attachment to VK2 vaginal epithelial cells (Fig. 3B), and we could visualize this difference via Gram staining (Fig. 3C and D). Further, the Fg adhesin mutant was also less adherent to Ect1 and End1 cervical epithelial cells (Fig. 3E and F). To assess the impact of these important surface adhesins during *in vivo* colonization, we cochallenged mice with WT USA300 and the Fg adhesin mutant. Initially, we recovered similar CFU of the two strains from the mice. However, by 3 days postinoculation, mice were significantly less colonized by the Fg adhesin mutant than by WT USA300 (Fig. 3G). At 5 days postinoculation, we could recover WT USA300 CFU from 60% of the mice, while only 30% were still colonized by the Fg adhesin mutant (Fig. 3G).

### Transcriptome analysis during MRSA vaginal colonization.

**FIG 4:**
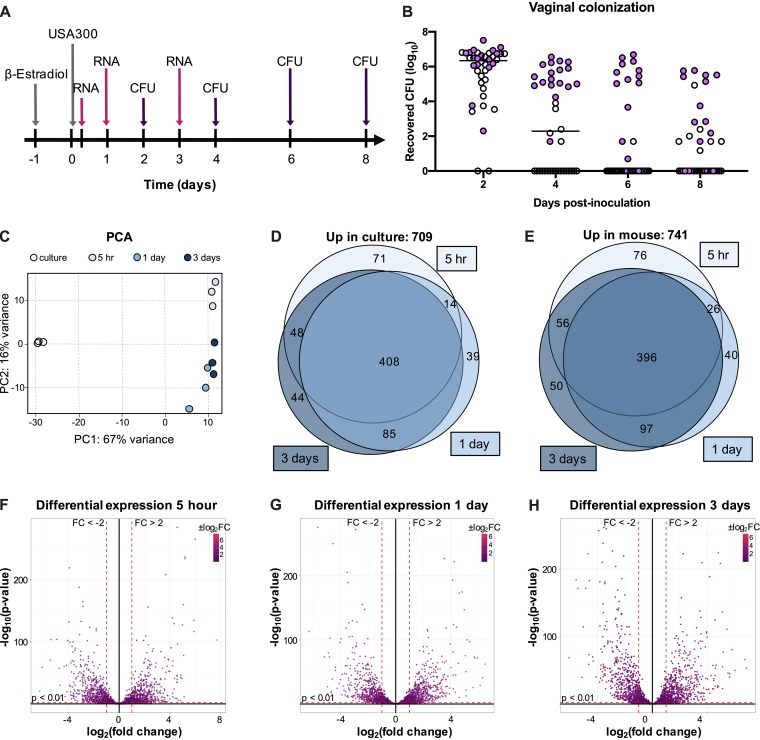
Transcriptome analysis during MRSA vaginal colonization. (A) Experimental design for RNA sequencing analysis of mouse vaginal swabs. (B) CFU counts from mouse vaginal swabs. Samples chosen for RNA sequencing are highlighted in purple. (C) PCA plot for triplicate samples of culture and 5 h, 1 day, and 3 day swabs. (D and E) Venn diagrams showing genes expressed at significantly higher levels (fold change, >2; *P* < 0.01) in culture (D) or in mouse swab samples (E). (F to H) Volcano plots highlighting genes that are differentially expressed in swab samples from 5 h (F), 1 day (G), and 3 days (H) postinoculation compared to culture.

Although the Fg adhesin mutant was impaired in vaginal persistence compared to WT USA300, we did not observe a significant difference in recovered CFU between the two strains during the first 2 days of colonization, and a few mice remained colonized with the Fg adhesin mutant at later time points (Fig. 1G). Thus, we hypothesized that other bacterial factors are involved in promoting MRSA vaginal carriage. To determine the impact of vaginal colonization on MRSA gene expression, we performed transcriptome analysis by RNA sequencing of USA300 recovered from the mouse vagina compared to USA300 cultured under laboratory conditions. For these experiments, we utilized the CD-1 mouse strain, as it is likely that in this background the bacteria encounter more host pressure to maintain colonization. Mice were pretreated with 17β-estradiol, inoculated with 10^7^ CFU of USA300, and swabbed at 5 h, 1 day, and 3 days postinoculation for RNA isolation. The same mice were swabbed 2, 4, 6, and 8 days postinoculation for CFU enumeration (Fig. 4A and B). Based on swab CFU counts, we selected samples from 18 mice (purple circles) for RNA sequencing analysis (Fig. 4B). RNA samples from 6 mouse swabs were pooled to generate 3 replicates for each time point to compare to triplicate culture samples. Principal-component analysis (PCA) for all of the samples showed that culture samples clustered separately from the mouse samples (Fig. 4C). Next, we compared mouse samples from each time point to the culture samples and observed that 709 genes were significantly upregulated in culture (Fig. 4D), and 741 genes were significantly upregulated (Fig. 4E) in the mouse (Table S1). Volcano plots of the log_2_(fold change) versus –log_10_(*P* value) show that many of the differentially upregulated and downregulated changes were highly significant at all three time points compared to culture (Fig. 4F to H). We observed significant overlap in differentially expressed transcripts at the various time points; over half of the differentially upregulated and downregulated genes were the same at all three time points (Fig. 4D and E).

10.1128/mBio.02321-19.3 TABLE S1 Differentially expressed genes in mouse swabs. All genes that were significantly differentially expressed in mouse samples are listed along with their adjusted *P* values, fold changes relative to culture in TSB, and absolute confidence. Download Table S1, XLSX file, 0.3 MB. Copyright © 2019 Deng et al. 2019 Deng et al. This content is distributed under the terms of the Creative Commons Attribution 4.0 International license.

We identified genes encoding transcriptional regulators, toxins, extracellular enzymes, and extracellular matrix-binding surface proteins that were significantly upregulated in the mouse at all three time points (Table 1). Interestingly, while only one immune evasion factor, chemotaxis inhibitor (*chs*), was upregulated at all three time points, additional immune evasion genes were significantly upregulated at 3 days postinoculation. We observed a similar trend with genes encoding components of the type VII secretion system (T7SS), which has been shown to contribute to S. aureus virulence and competition with other microbes in polymicrobial settings (53, 54). At 5 h postinoculation, only 5 T7SS genes were significantly upregulated, while 14 genes were upregulated at 1 day and 3 days postinoculation (Table 1).

**TABLE 1:** Virulence factors which were significantly upregulated during vaginal colonization^*a*^

Category	Locus tag	Gene	Fold change by time postinoculation		
			5 h	1 day	3 days
Transcriptional regulation	SAUSA300_0195	Transcriptional regulator	3	3	3
	SAUSA300_0218	Sensor histidine kinase family protein	3	3	4
	SAUSA300_0255	Two-component system response regulator	6	7	6
	SAUSA300_0878	LysR family transcriptional regulator	9	5	5
	SAUSA300_1220	LuxR family DNA-binding response regulator	2	2	2
	SAUSA300_1257	*msrR*	5	5	4
	SAUSA300_1514	*fur*	3	3	2
	SAUSA300_1717	*arsR*	7	4	5
	SAUSA300_1798	DNA-binding response regulator	4	4	4
	SAUSA300_1799	Putative sensor histidine kinase	3	4	4
	SAUSA300_2098	Transcriptional repressor	15	13	15
	SAUSA300_2300	TetR family transcriptional regulator	3	3	3
	SAUSA300_2322	TetR family transcriptional regulator	4	4	4
	SAUSA300_2336	MerR family transcriptional regulator	3	2	3
	SAUSA300_2347	*nirR*	12	8	7
	SAUSA300_2437	*sarT*	11	18	13
	SAUSA300_2566	*arcR*	4	4	6
	SAUSA300_2571	*argR*	3	4	6
	SAUSA300_2640	Putative transcriptional regulator	5	9	11
Toxins	SAUSA300_0800	*sek*	6	7	7
	SAUSA300_0801	*seq*	4	6	5
	SAUSA300_1058	*hla*	19	6	18
	SAUSA300_1918	Truncated beta-hemolysin	14	2	9
Secreted enzymes	SAUSA300_0923	*htrA*	3	4	4
	SAUSA300_0951	*sspA*	2	12	13
	SAUSA300_1753	*spIF*	12	3	24
	SAUSA300_1755	*spID*	13	5	28
	SAUSA300_1756	*spIC*	11	3	22
	SAUSA300_1757	*spIB*	12	4	21
	SAUSA300_2572	*aur*	4	15	12
ECM binding	SAUSA300_0546	*sdrC*	6	12	16
	SAUSA300_0547	*sdrD*	9	19	22
	SAUSA300_0774	*empbp*	35	4	8
Immune modulation	SAUSA300_1059	Superantigen-like protein	10	3	4
	SAUSA300_1060	Superantigen-like protein	12	4	6
	SAUSA300_1061	Superantigen-like protein	8	6	7
	SAUSA300_1920	*chs*	29	3	8
	SAUSA300_0224^*b*^	*coa*			4
	SAUSA300_0836^*b*^	*dltB*			2
	SAUSA300_0837^*b*^	*dltC*			2
	SAUSA300_1053^*b*^	Formyl peptide receptor-like 1 inhibitory protein			4
	SAUSA300_1055^*b*^	*efb*			2
	SAUSA300_2364^*b*^	*sbi*			2
Type 7 secretion system	SAUSA300_0279^*b*^	*esaA*			5
	SAUSA300_0280^*b*^	*essA*			2
	SAUSA300_0281^*b*^	*esaB*		4	4
	SAUSA300_0282	*essB*	2	6	6
	SAUSA300_0283^*b*^	*essC*		3	3
	SAUSA300_0284^*b*^	*esxC*		3	2
	SAUSA300_0286^*b*^	*essE*		3	3
	SAUSA300_0287^*b*^	*esxD*		3	3
	SAUSA300_0288^*b*^	*essD*		2	
	SAUSA300_0290	DUF5079 family protein	6	9	9
	SAUSA300_0291	DUF5080 family protein	5	7	6
	SAUSA300_0298	*essI5*	3	4	3
	SAUSA300_0299^*b*^	*essI6*		2	
	SAUSA300_0300^*b*^	*essI7*		2	2
	SAUSA300_0302	*essI9*	3	5	5
	SAUSA300_0303^*b*^	DUF4467 domain-containing protein		2	3

a Genes which encode transcriptional regulators, toxins, secreted enzymes, extracellular matrix (ECM) binding, immune modulation, and T7SS factors and that were differentially expressed in mouse swab samples at all three time points are listed with their respective fold changes relative to growth in TSB.

b Genes which were not significantly differentially expressed at all time points.

### Iron homeostasis impacts vaginal persistence.

**FIG 5:**
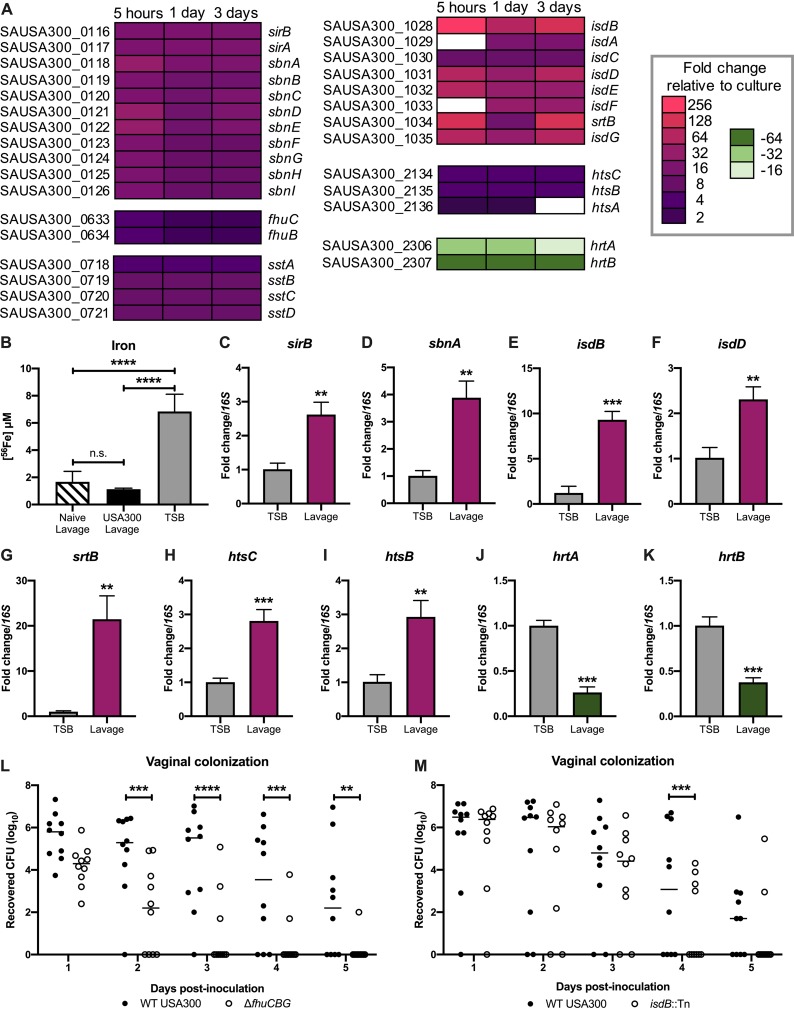
Iron homeostasis impacts vaginal persistence. (A) Differential expression of genes in iron acquisition and iron export pathways. (B) ICP-MS analysis of vaginal lavage fluid from naive and colonized mice (*n* = 3 per group) and TSB. Error bars represent SDs. (C to K) RT-qPCR confirmation of select RNA sequencing iron homeostasis hits. (L) Cocolonization with WT USA300 and the Δ*fhuCBG* mutant. (M) Cocolonization with WT USA300 and the *isdB*::Tn mutant. Statistical analysis using one-way ANOVA (B), unpaired *t* test (C to K), and two-way ANOVA with Sidak’s multiple-comparison test (L and M). **, *P* < 0.005; ***, *P* < 0.0005; ****, *P* < 0.00005.

Though there were global transcriptional changes, the most highly significant, differentially expressed transcripts belonged to iron uptake and export systems. The most highly induced was the iron surface determinant *isd* heme acquisition system (*isdBACDEFG* and *srtB*). Other genes included those involved in the production of the siderophore staphyloferrin B (SB) (*sbnABCDEFGHI*), as well as its importer (*sirAB*), the staphyloferrin A (SA) importer (*htsABC*), the xeno-siderophore transporter (*fhuCB*), as well as the catechol/catecholamine iron transporter system (*sstABCD*). Lastly, the heme-regulated export *hrt* system was highly downregulated during colonization (*hrtAB*) (Fig. 5A) (55–58). As these results strongly suggest that the vaginal environment is iron limited, we performed inductively coupled plasma mass spectrometry (ICP-MS) to determine the iron concentration in vaginal lavage fluid from naive mice and mice colonized with USA300. We observed a very low concentration of iron (1.4 μM), irrespective of MRSA colonization, compared to the level present in tryptic soy broth (TSB) (6.8 μM) (Fig. 5B).

To confirm the differential expression of iron uptake systems by USA300, we incubated USA300 in mouse vaginal lavage fluid and performed reverse transcription-quantitative PCR (RT-qPCR) to compare the transcripts of select iron homeostasis genes between bacteria grown in lavage fluid and bacteria cultured under laboratory conditions in TSB. Similar to our RNA sequencing (RNA-seq) results, the RT-qPCR analysis revealed an increase in *sirB*, *sbnA*, *isdB*, *isdD*, *srtB*, *htsC*, and *htsB* transcripts in MRSA cultured in vaginal lavage fluid (Fig. 5C to I). Additionally, *hrtA* and *hrtB* were significantly downregulated in MRSA from vaginal lavage fluid compared to MRSA grown in TSB (Fig. 5J and K).

To assess the impact of iron uptake by MRSA on vaginal persistence, we cocolonized mice with WT USA300 and either the Δ*fhuCBG* or *isdB*::Tn mutant. In addition to its role in the uptake of xeno-siderophores, the FhuC ATPase also provides the energy needed for uptake of the siderophores SA and SB. Therefore, the Δ*fhuCBG* mutant is defective in the transport of all siderophores (59). Also, our RNA-seq results show that at all three time points, the most highly upregulated gene was *isdB*, which encodes the hemoglobin-binding surface protein that transports heme to downstream components of the *isd* system (56). *isdB* transcripts from mouse samples were increased 210-fold at 5 h, 90-fold at 1 day, and 117-fold at 3 days postinoculation compared to those in culture (Table S1 and Fig. 5A). Compared to WT USA300, the Δ*fhuCBG* mutant and the *isdB*::Tn mutant were cleared significantly faster from the mouse vagina (Fig. 5L and M). Because a previous study reported that IsdB may impact bacterial attachment to host cells (60), we quantified adherence of the *isdB*::Tn mutant to VK2, Ect1, and End1 cells *in vitro* and observed no defect compared to WT USA300 (Fig. S2A to C).

10.1128/mBio.02321-19.2 FIG S2 (A to C) WT USA300 and *isdB*::Tn adherence to VK2 (A), End1 (B), and Ect1 (C) cells. Experiments were performed at least three times in triplicate, and error bars represent standard deviations (SDs); results from a representative experiment are shown. (D) Growth of WT USA300, *isdB*::Tn mutant, and *isdB*::Tn/pCM28*isdB*-complemented strains in CDM supplemented with 40 nM hemoglobin. Absorbance at 600 nm was measured at 6 hours. The experiments were performed in biological triplicates with technical duplicates, and the error bars represent SDs. Statistical analysis was performed using a one-way ANOVA with Tukey’s multiple-comparison test. *, *P* < 0.05; **, *P* < 0.005. Download FIG S2, TIF file, 0.2 MB. Copyright © 2019 Deng et al. 2019 Deng et al. This content is distributed under the terms of the Creative Commons Attribution 4.0 International license.

## DISCUSSION

S. aureus is capable of causing disease in nearly every site of the body (61), and MRSA colonization of the skin and mucosal sites, such as the nares and the vaginal tract, is a necessary initial step preceding the development of invasive disease (27, 37, 62–64). While many studies have investigated host and bacterial determinants of S. aureus colonization of the skin and nares as well as subsequent infection, little is known about factors which influence vaginal niche establishment and persistence. Because vaginal carriage during pregnancy represents a major risk factor for the transmission of this pathogen to the newborn (24, 25, 65, 66), we utilized *in vitro* and *in vivo* models of MRSA vaginal colonization to identify determinants of persistence within the female reproductive tract. The results of our study reveal that MRSA can interact directly with the female reproductive tract epithelium *in vitro* and *in vivo*, and that the expression of cell wall-anchored Fg binding adhesins as well as iron acquisition systems promote MRSA vaginal colonization.

The murine MRSA vaginal colonization protocol developed in this study is comparable to the well-established models of mouse and cotton rat nasal S. aureus carriage; animals are inoculated with a similar dose, between 10^7^ and 10^8^ CFU of bacteria, and remain colonized for several days postinoculation. However, one key difference between the vaginal colonization model and the models of nare colonization is that MRSA vaginal persistence can be sampled in the same mice multiple times during the course of one experiment. In contrast, because cotton rats and mice must be euthanized in order to assess nasal carriage of S. aureus, the animals can be sampled at only one time point during the course of the experiment (67–69).

The effect of S. aureus colonization on the host immune response has been well characterized at many epithelial sites. S. aureus on the skin promotes a robust inflammatory response involving both the innate and adaptive immune system (70, 71). Neutrophils in particular are rapidly and highly recruited to the site of S. aureus skin infection and are key mediators of clearance of the pathogen (72–79). Our studies on GBS vaginal carriage have shown a clear role for neutrophils in combating GBS colonization of this host site (44, 80). Additionally, neutrophils have been shown to respond to other common pathogens of the vaginal tract, such as the fungus Candida albicans (81, 82) and the Gram-negative bacterium Neisseria gonorrhoeae (83). In this study, we observed an increased neutrophil presence in the vaginal tissues from mice colonized by MRSA compared to naive controls. Interestingly, while there is obvious neutrophil infiltration of the lamina propria of the vagina 1 day postcolonization with MRSA, we did not detect neutrophils in the vaginal lumen at this early time point. In contrast, at 3 days postinoculation, we could visualize many neutrophils within the vaginal lumen. The timing of the infiltration of neutrophils into the vaginal lumen coincides with the increased expression of immune evasion factors by MRSA; in our RNA sequencing analysis, we observed significant upregulation of these factors at 3 days postinoculation and not at earlier time points. Future studies aimed at further characterizing the dynamics of the neutrophil response to MRSA in the female reproductive tract and their extravasation into the vaginal lumen may reveal new insights into host immune responses common to all vaginal pathogens as well as those specific to MRSA.

The impact of S. aureus interactions with Fg on colonization and disease at various tissue sites has been well characterized. In the context of invasive infections, Fg and fibrin can promote clearance of S. aureus by containing the bacteria within aggregates (84, 85). Additionally, Fg can stimulate the production of inflammatory cytokines and activate neutrophils (86–88). However, S. aureus has also been shown to target Fg to promote persistence and disease in the host. The bacterium can interact with Fg in order to coagulate or to form clumps which help it evade immune detection, and this clumping is mediated by surface Fg binding adhesins, including ClfA and ClfB (47, 89–92). There is also evidence that S. aureus can alter gene regulation in the presence of fibrinogen-containing clumps to enhance the expression of virulence determinants (93). Moreover, S. aureus can use Fg as part of its biofilm structure to promote persistence within the host (94). Our data suggest that, in the context of vaginal colonization, MRSA interactions with Fg are necessary for persistence within the host. A mutant deficient in Fg binding was significantly impaired in its ability to adhere to human female reproductive tract cells *in vitro* and was also rapidly cleared from the vaginal tract *in vivo* compared to the WT. These results hint that the benefits of MRSA binding to Fg outweigh the potential detriments for the pathogen during vaginal colonization.

While a majority of mice rapidly clear the Fg adhesin mutant during vaginal colonization, it is able to persist in some of the animals (Fig. 3G). This result suggests that there are likely other factors that contribute to *in vivo* vaginal colonization. To identify additional determinants of vaginal persistence, we performed RNA sequencing to profile the transcriptome of MRSA during vaginal colonization. We observed that over one-quarter of the genes of USA300 were differentially expressed during *in vivo* colonization, and over half of those genes were differentially expressed at all three *in vivo* time points that were analyzed. Of note, many of the most highly and significantly differentially expressed genes belonged to iron acquisition or iron homeostasis pathways. Our observation that genes involved in iron uptake were upregulated was not surprising since their expression is controlled by iron levels, and our ICP-MS data revealed the vaginal environment to be limited in iron (Fig. 5B). Using our *in vivo* murine vaginal colonization model, we confirmed that mutants in *fhuCBG* and *isdB* exhibited decreased persistence compared to the isogenic WT MRSA strain. Numerous reports have demonstrated the importance of nutrient iron for S. aureus growth and pathogenicity (56, 95, 96), and the results of our study highlight the necessity of this metal for MRSA colonization and persistence within the vaginal environment. That the Δ*fhuCBG* mutant was attenuated in this model was interesting because, while FhuCBG is known to transport hydroxamate-type siderophores which S. aureus does not synthesize (58, 97), FhuC is also the ATPase which provides energy for the uptake of both SA and SB siderophores (56, 59). Both the WT and the Δ*fhuCBG* mutant should, under the iron-restricted conditions during vaginal colonization, express SA and SB. Given that the Δ*fhuCBG* mutant cannot transport these siderophores, the extracellular environment becomes more iron restricted to the mutant, as it cannot access SA-Fe and SB-Fe chelates.

The limitation of iron is a major host mechanism for defending against pathogens because this metal is vital for bacterial growth and metabolic processes (56, 98, 99). Other transcriptomic studies examining S. aureus growing *in vivo* during invasive infections have shown that the bacteria respond to nutrient limitation within the host. One study which compared the transcriptomes of S. aureus in a murine osteomyelitis model to bacteria grown under laboratory conditions revealed the importance of iron homeostasis mechanisms, especially the Isd pathway, during chronic infection (100). Another analysis of USA300 gene expression during human and mouse infections also showed upregulation of iron transporters *in vivo* (101). Interestingly, many reports have shown that neutrophils can play an active role in limiting iron in numerous host sites, including the vagina, during exposure to a bacterial pathogen (102–105). The precise mechanisms by which the host restricts iron availability during colonization warrants further research, as this would provide insight into the exact function of neutrophils in controlling MRSA vaginal persistence.

We have developed a murine model of S. aureus vaginal colonization, and this study is the first to investigate the molecular mechanisms that promote vaginal carriage and persistence by MRSA. This mouse model will be useful for continued studies on MRSA-host interactions within a mucosal environment. Here, we demonstrate the importance of Fg binding and iron acquisition in promoting long-term colonization. Additionally, we observed that neutrophils respond to the presence of MRSA in the vagina and that the bacteria upregulate the expression of immunomodulating genes during the course of colonization. Further investigation into these specific colonization determinants could yield therapeutic interventions to treat MRSA persistence within this host niche.

## MATERIALS AND METHODS

### Bacterial strains and culture conditions.

S. aureus strains USA300 (39) and MRSA252 (40) were used for the experiments. S. aureus was grown in tryptic soy broth (TSB) at 37°C, and growth was monitored by measuring the optical density at 600 nm (OD_600_). For selection of S. aureus mutants, tryptic soy agar (TSA) was supplemented with chloramphenicol (Cm) (10 μg/ml), erythromycin (Erm) (3 μg/ml), or tetracycline (Tet) (1 μg/ml).

To generate the Fg adhesin mutant, first the *fnbAB* operon was deleted using allelic replacement. Phage 80α or 11 was used for transduction between S. aureus strains (108). The *fnbAB* markerless deletion plasmid pHC94 was constructed using Gibson assembly with the plasmid backbone coming from the amplification of pJB38 (109) using primers pJB38 rev2 and pJB38 fwd2. The region upstream of *fnbA* was amplified with primers fnbAB delA and fnbAB delB, and the region downstream of *fnbB* was amplified using fnbAB delC and fnbAB delD (Table S2). The resulting plasmid was electroporated in S. aureus RN4220 (110), with selection on TSA Cm plates at 30°C. The plasmid was then transduced into S. aureus strain LAC Δ*clfA* (89). Individual colonies were streaked on TSA Cm plates incubated at 42°C to select for integration of the plasmid into the chromosome. Single colonies were grown in TSB at 30°C and reinoculated into fresh medium for several days before plating on TSA containing anhydrotetracycline (0.3 μg/ml) to select for loss of the plasmid, creating the LAC Δ*clfA* Δ*fnbAB* mutant. The *clfB*::Tn mutation was than transduced into this background from the Nebraska Transposon Mutant Library (111) and selected on TSA Erm plates. The Fg adhesin mutant grew similarly to the parental WT strain in TSB.

10.1128/mBio.02321-19.4 TABLE S2 List of primers used in this study. Download Table S2, XLSX file, 0.01 MB. Copyright © 2019 Deng et al. 2019 Deng et al. This content is distributed under the terms of the Creative Commons Attribution 4.0 International license.

The *isdB mariner*-based transposon *bursa aurealis* mutation (JE2 *isdB*::ΦNΣ, NE1102) from the Nebraska Transposon Library (111) was transferred into USA300 LAC with phage 11, as described previously (112). S. aureus genomic DNA of LAC* *isdB*::ΦNΣ (USA300 *isdB*::Tn) was isolated using the Puregene DNA purification kit (Qiagen), and the transposon insertion was verified by PCR with primers KAS249 and KAS250 (Table S2). The USA300 *isdB*::Tn mutant was complemented with a copy of the *isdB* gene (SAUSA300_1028) expressed on a plasmid. A 2,280-bp fragment, containing the *isdB* gene and its native promoter, was amplified from LAC chromosomal DNA using primers KAS276 and KAS277 (Table S2). The product was digested with BamHI and SalI and ligated into the same restriction sites in pCM28 (119). The resulting plasmid was electroporated into Escherichia coli DC10B (113), and sequencing was performed at the Molecular Biology Service Center at the University of Colorado Anschutz Medical Campus with chromosomal and vector primers KAS278, KAS113, KAS116, KAS249, KAS265, and KAS277 (Table S2). The empty vector (pCM28) and the complementation vector (pCM28*isdB*) were then electroporated into USA300 *isdB*::Tn. The *fhuCBG* and *isdB*::Tn mutants grew similarly to the parental WT strain in TSB. As expected, the *isdB*::Tn mutant exhibited a growth deficiency in iron-depleted medium supplemented with 40 nM hemoglobin that was complemented by plasmid expression of *isdB* (Fig. S2D).

As we have previously reported in reference 59, S. aureus lacking the *fhuCBG* genes exhibits a growth deficiency in iron-limited medium, and this defect can be complemented by expressing just *fhuC* on a plasmid. The Δ*fhuCBG* mutation was transferred into USA300 LAC with phage 11, as described previously (112), and confirmed to have a similar phenotype that was complemented (data not shown). The DsRed expressing USA300 (114) and WT USA300 carrying the pCM28 plasmid were generated previously (115).

### *In vitro* MRSA adherence assays.

Immortalized VK2 human vaginal epithelial cells, Ect1 human ectocervical endothelial cells, and End1 human endocervical epithelial cells were obtained from the American Type Culture Collection (VK2.E6E7, ATCC CRL-2616; Ect1/E6E7, ATCC CRL-2614; and End1/E6E7, ATCC CRL-2615) and were maintained in keratinocyte serum-free medium (KSFM; Gibco) with 0.1 ng/ml human recombinant epidermal growth factor (EGF; Gibco) and 0.05 mg/ml bovine pituitary extract (Gibco) at 37°C with 5% CO_2_.

Assays to determine cell surface-adherent MRSA were performed as described previously (41). Briefly, bacteria were grown to mid-log phase to infect cell monolayers (multiplicity of infection [MOI], 1). After a 30-min incubation, cells were detached with 0.1 ml of a 0.25% trypsin-EDTA solution and lysed with the addition of 0.4 ml of 0.025% Triton X-100 by vigorous pipetting. The lysates were then serially diluted and plated on TSA to enumerate the bacterial CFU. Experiments were performed at least three times under each condition in triplicate, and results from a representative experiment are shown as indicated in the figure legends.

Crystal violet fibrinogen adhesion assays were performed as described in reference 89. Briefly, 96-well plates (Corning) were coated with 20 μg/ml human fibrinogen and incubated with 100 μl of bacterial suspensions in phosphate-buffered saline (PBS) at an OD_600_ of 1.0 for 1 h at 37°C. The wells were then washed and dried, and the adherent bacteria were stained with 0.1% crystal violet. The bound crystal violet stain was solubilized with 33% acetic acid and the OD_570_ measured.

For Gram staining analysis, VK2 monolayers were grown in tissue culture-treated chamber slides (Thermo Fisher) and infected with either WT USA300 or the fibrinogen adhesin mutant at an MOI of 20. Following a 30-min incubation, the cell monolayers were washed to remove any nonadherent bacteria and then fixed with 10% formalin (Fisher) and Gram stained (Sigma).

### Murine vaginal colonization model.

Animal experiments were approved by the Institutional Animal Care and Use Committee at the University of Colorado-Anschutz Medical Campus under protocol number 00316 and performed using accepted veterinary standards. A mouse vaginal colonization model for GBS was adapted for our studies (38). Eight-week-old female CD-1 (Charles River), C57BL/6 (Jackson), and BALB/c (Jackson) mice were injected intraperitoneally with 0.5 mg of 17β-estradiol (Sigma) 1 day prior to colonization with MRSA. Mice were vaginally inoculated with 10^7^ CFU of MRSA in 10 μl PBS, and on subsequent days, the vaginal lumen was swabbed with a sterile ultrafine swab (Puritan). To assess the tissue CFU, mice were euthanized according to approved veterinary protocols, and the female reproductive tract tissues were placed into 500 μl PBS and bead beaten for 2 min to homogenize the tissues. The recovered MRSA bacteria were serially diluted and enumerated on CHROMagar (Hardy Diagnostics) supplemented with 5.2 μg/ml cefoxitin.

### Histology.

Mouse female reproductive tract tissues were harvested and embedded into O.C.T. compound (Sakura) and sectioned with a CM1950 freezing cryostat (Leica). For fluorescence microscopy, coverslips were mounted with Vectashield mounting medium with 4′,6-diamidino-2-phenylindole (DAPI; Vector Labs). H&E staining was performed using reagents from Sigma. Immunohistochemical analysis was performed using a biotinylated primary antibody against Gr-1 (BioLegend), streptavidin conjugated to horseradish peroxidase (Jackson Immunoresearch), and a 3-amino-9-ethylcarbazole (AEC) peroxidase substrate kit (Vector Labs). Images were taken with a BZ-X710 microscope (Keyence).

### Generation of RNA sequencing data.

USA300 (10^7^ CFU) was inoculated into the mouse vagina, and mice were swabbed vaginally at 5 h, 1 day, and 3 days postinoculation for RNA recovery. Vaginal swabs were placed into TRIzol reagent (Thermo Fisher), vortexed to dissociate the bacteria from the swabs, and stored at –80°C. Swab samples from 6 mice were pooled, and bacteria were lysed by beating for 2 min at maximum speed on a bead beater (BioSpec Products). RNA was isolated by following the manufacturer’s protocol using a Direct-zol RNA MiniPrep Plus kit (Zymo Research). For each sample, 120 ng total RNA was ribodepleted using the Ribo-Zero magnetic gold kit (Epidemiology) from Epicentre (Illumina), following the manufacturer’s protocol. Ribodepleted RNA was then prepared into sequence libraries using the RNA Ultra II kit (New England BioLabs), following the manufacturer’s protocol without fragmentation. Libraries underwent 9 cycles of PCR before 1× AMPure bead purification (Beckman Coulter). Libraries were quantified, pooled, and sequenced on an Illumina NextSeq 500 platform with 75-base single reads targeting 20 million reads per samples.

### Analysis of RNA sequencing data.

Sequencing reads were aligned to the NCBI reference sequence with GenBank accession number NC_007793.1, and expression levels were calculated using Geneious 11.1.5. Transcripts with an adjusted *P* value of <0.05 and log_2_ fold change of ±1 were considered significantly differentially expressed. PCA and volcano plots were generated using the ggplot2 package in R. Venn diagrams were generated using the area-proportional Venn diagram tool (BioInfoRx).

### ICP-MS analysis.

Naive and S. aureus-colonized female CD1 mice (*n* = 3/group) were lavaged at 24 h postinoculation two times with 50 μl sterile PBS. The lavage fluid was diluted 1:10 in PBS and filtered through 0.22-μm Spin-X centrifuge tube filters (Costar). Vaginal lavage fluid and medium samples were diluted 1:20 and analyzed on an Agilent 7500cx ICP-MS at the University of Nebraska Spectroscopy and Biophysics Core.

### RT-qPCR confirmation of RNA sequencing.

Vaginal lavage fluid was collected as described in reference 38 and filtered through 0.22-μm Spin-X centrifuge tube filters (Costar) to remove contaminants. Triplicate log-phase cultures of USA300 were pelleted and resuspended in filtered lavage fluid. Following a 2-h incubation at 37°C, bacteria were collected by centrifugation, resuspended in TRIzol, and lysed by bead beating, and RNA was isolated using the Direct-zol RNA MiniPrep Plus kit, as described above. RNA was treated with Turbo DNase (Invitrogen) to remove contaminating DNA. cDNA was generated using the Quanta cDNA synthesis kit (Quanta Biosciences), and qPCR was performed using PerfeCTa SYBR green reagent (Quanta) and a CFX96 real-time PCR thermal cycler (Bio-Rad). Fold changes were calculated using the Livak method (116).

### Growth in iron-depleted medium supplemented with hemoglobin.

Successful complementation of the *isdB*::Tn mutant was tested by using a modified protocol from references 117 and 118. S. aureus cultures were grown overnight in RPMI 1640 medium (Thermo Fisher) supplemented with 1% Casamino Acids (BD Biosciences) plus 400 μM 2,2′-bipyridine. Overnight cultures were centrifuged (2,800 × *g*, 10 min), and the pellets were resuspended in NRPMI^+^ (Chelex-treated RPMI containing 500 μM 2,2′-bipyridine and 25 μM ZnCl_2_, 25 μM MnCl_2_, 100 μM CaCl_2_, and 1 mM MgCl_2_). All cultures were set to an OD_600_ of 0.02 in NRPMI^+^ without and with 40 nM hemoglobin (hemoglobin human; Sigma). Bacterial growth (absorbance at 600 nm) was monitored using a Synergy H1 microplate reader set to 37°C with continuous shaking (548 cpm). The experiments were performed in biological triplicates with technical duplicates.

### Data analysis.

GraphPad Prism version 7.0 was used for statistical analysis, and statistical significance was accepted at *P* values of <0.05 (*, *P* < 0.05; **, *P* < 0.00005; ***, *P* < 0.0005; **** *P* < 0.00005). Specific tests are indicated in figure legends.
