# Supplementary figures and images for: Identification of Key Determinants of Staphylococcus aureus Vaginal Colonization
Source: mBio. 2019 Dec 24;10(6):e02321-19. doi: 10.1128/mBio.02321-19 (PMC6935855; doi:10.1128/mBio.02321-19)

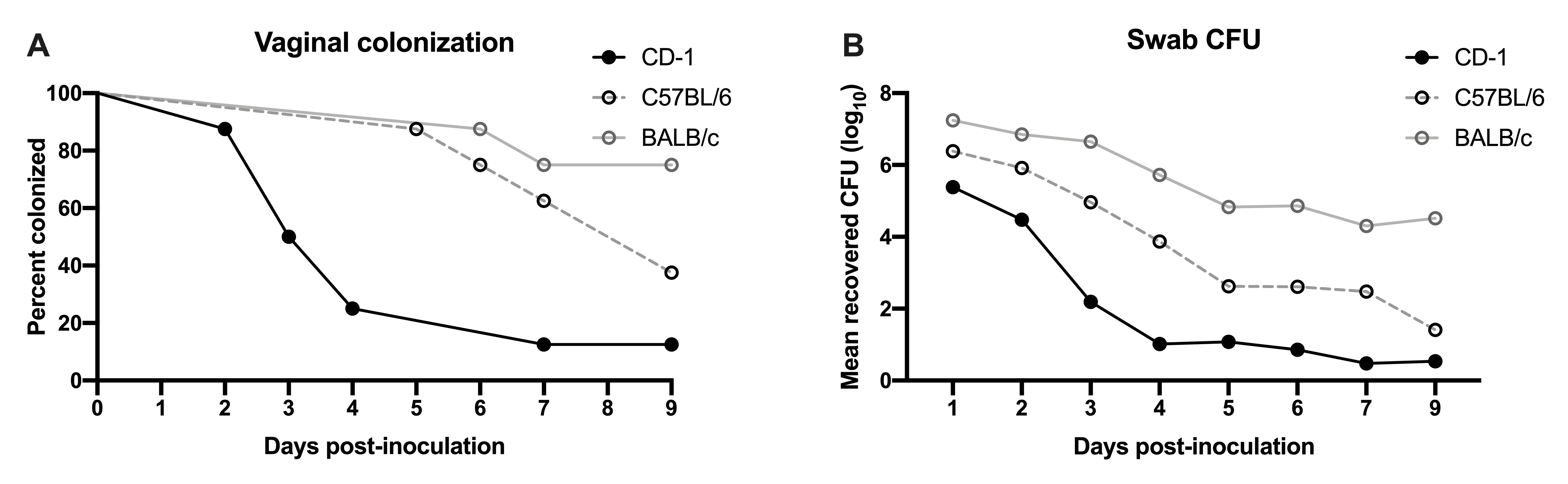

Supplement: FIG S1 [file mBio.02321-19-sf001.tif]

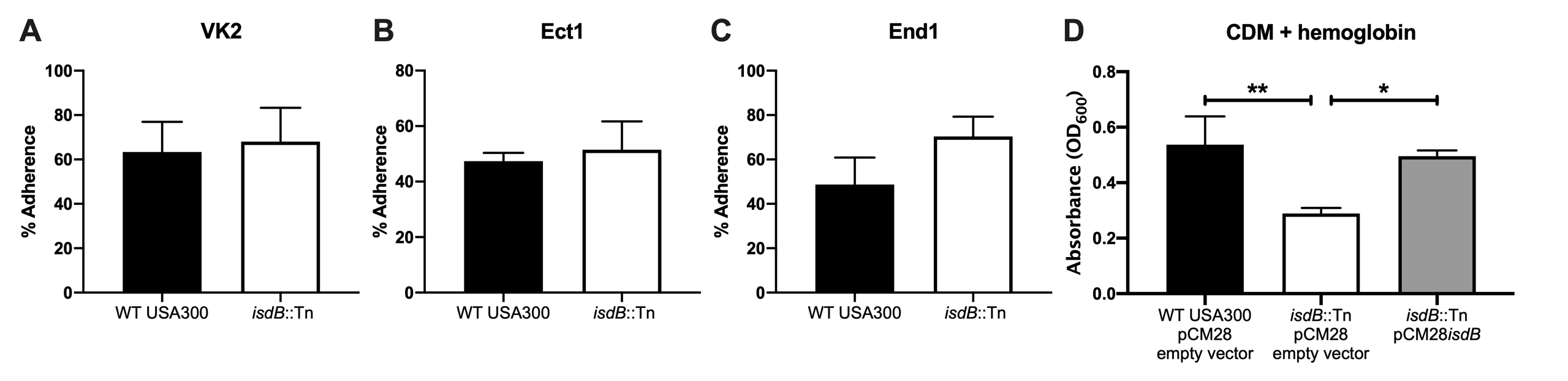

Supplement: FIG S2 [file mBio.02321-19-sf002.tif]
